# A Rare Manifestation of a Rare Disease: Mantle Cell Lymphoma Presenting With Aseptic Meningitis

**DOI:** 10.1177/2324709619858643

**Published:** 2019-06-24

**Authors:** Elvira Umyarova, Sreedhar Adapa, Srikanth Naramala, Vijay Gayam, Narothama Reddy Aeddula, Venu Madhav Konala

**Affiliations:** 1 University of Vermont, Burlington, VT, USA; 2 Kaweah Delta Medical Center, Visalia, CA, USA; 3 Adventist Medical Center Hanford, Hanford, CA, USA; 4 Interfaith Medical Center, Brooklyn, NY, USA; 5 Deaconess Health System Inc, Evansville, IN, USA; 6 Ashland-Bellefonte Cancer Center, Ashland, KY, USA

**Keywords:** mantle cell lymphoma, aseptic meningitis, MIPI

## Abstract

Mantle cell lymphoma (MCL) is a rare form of non-Hodgkin lymphoma characterized by clonal proliferation of follicular mantle zone B lymphocytes. It is caused by abnormal chromosomal translocation t(11;14) resulting in aberrant expression of cyclin D1. This leads to activation of anti-apoptotic pathways and abnormal proliferation of MCL cells. Patients can present with an indolent course or a fulminant disease with short overall survival. The disease frequently involves extranodal organs, but rarely manifests with neurological symptoms. We report a rare case of aberrant CD5-negative MCL presenting with aseptic meningitis.

## Introduction

Mantle cell lymphoma (MCL) is a malignant tumor derived from B lymphocytes in the mantle zone of lymphoid follicles, which is characterized by specific morphologic, immunophenotypic, and molecular genetic features. MCL usually has an aggressive clinical course. General treatment involves aggressive chemo-immunotherapy in younger patients followed by autologous stem cell transplant and maintenance rituximab. In older patients, chemo-immunotherapy followed by maintenance rituxumab is the current standard of care.^1^ Initial therapy can be deferred in the patients who present with the more indolent course without adverse impact on survival. MCL often involves extranodal organs, particularly the bone marrow and gastrointestinal tract, but very rarely manifests solely with neurological symptoms.

## Case Presentation

A 31-year-old Hispanic male without any significant past medical history presented to the hospital with a 3-day history of altered mental status, flu-like symptoms, severe headache, and pancytopenia. Physical examination revealed mild neck stiffness, and the rest of the examination was unremarkable. Laboratory abnormality was significant for pancytopenia. The patient underwent lumbar puncture and was started on empiric antibiotics along with acyclovir. Cerebrospinal fluid (CSF) analysis showed elevated white blood cells (WBCs) with lymphocyte predominance and was negative for bacterial, viral, and fungal growth. The patient clinically improved and was discharged with a diagnosis of aseptic meningitis.

Two months later, he was readmitted to the hospital with similar symptoms. Results of repeated lumbar puncture with analysis of CSF showed elevated WBC with lymphocyte predominance as well as elevated protein. Further testing of CSF was negative for *Cytomegalovirus*, West Nile virus, herpes simplex virus, varicella zoster virus, as well as other bacterial and fungal pathogens. As part of further assessment, the patient underwent screening for HIV 1/2; hepatitis A, B, C; infectious mononucleosis; *Ehrlichia; Cryptococcus*; and parvovirus infection, which were all negative. Bone marrow biopsy was performed due to persistent pancytopenia.

**Figure 1.**
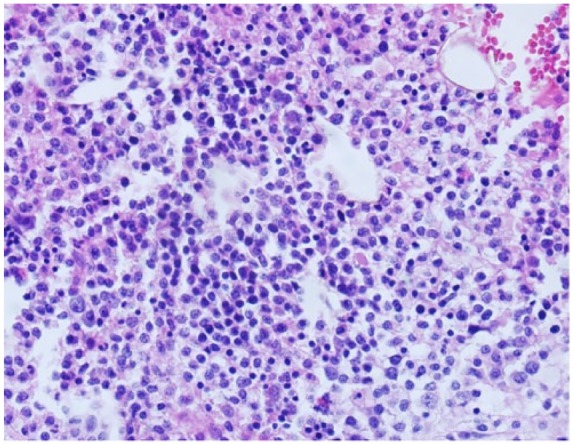
Bone marrow: morphology suspicious for a lymphoproliferative disorder.

**Figure 2.**
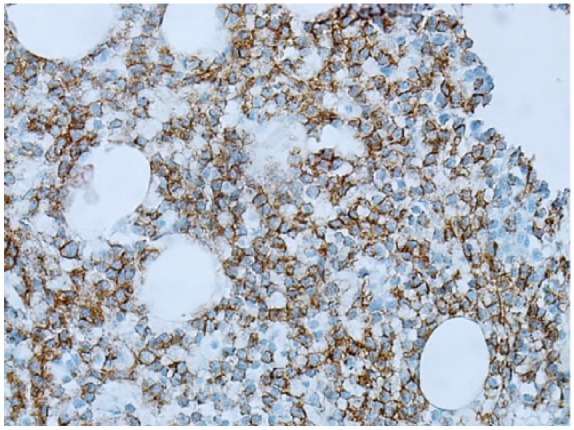
Bone marrow: CD20 staining.

**Figure 3.**
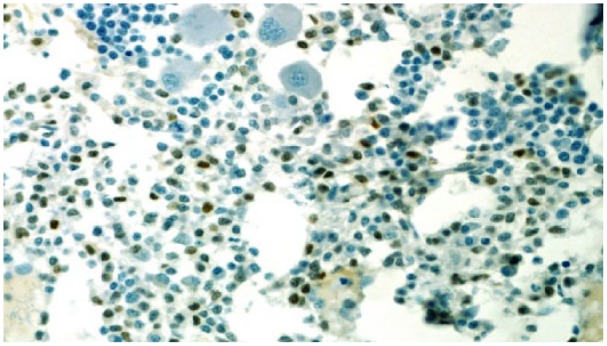
Bone marrow: cyclin D1 staining.

The results were suspicious for B-cell lymphoproliferative disorder (Figure 1), and the specimen was sent for a second opinion to Mayo Clinic. Morphological results revealed extensive atypical interstitial lymphocytic infiltrate, composed of small cells with round-to-slightly irregular nuclear contours. Immunohistochemical analysis of atypical lymphocytes was strongly positive for CD20 (Figure 2) without co-expression of CD5 or CD10. B-cell population had a lambda light chain restriction. Additional immunostains of atypical lymphocytes showed expression of BCL-2 protein and cyclin D1 (Figure 3). Results of bone marrow biopsy were consistent with rare aberrant CD5 negative MCL.

Clinical picture and results of bone marrow investigations raised a concern of primary central nervous system (CNS) involvement. The patient underwent magnetic resonance imaging of the brain with unremarkable results. Immunohistochemical analysis of CSF by flow cytometry revealed prominent monoclonal B-cell lymphoid population with immunohistochemical characteristics similar to the B-cells found on bone marrow biopsy. Further assessment with computed tomography scans of neck, chest, abdomen, and pelvis failed to reveal lymphadenopathy.

The patient received a total of 8 cycles of R-HyperCVAD (rituximab along with cyclophosphamide, vincristine sulfate, doxorubicin hydrochloride, and dexamethasone) alternating with rituximab, high-dose methotrexate and cytarabine (R-MA), and CNS prophylaxis with methotrexate. He responded well to treatment with rapid resolution of leucopenia and thrombocytopenia. His restaging computed tomography scans of the chest, abdomen, and pelvis did not show any signs of lymphadenopathy. Follow-up bone marrow biopsy was negative for lymphoma involvement and repeated CSF analysis showed no evidence of disease. The patient continues to be in complete response and is under active surveillance.

## Discussion

Mantle cell lymphoma is a rare subtype of B-cell non-Hodgkin’s lymphoma (NHL), which is characterized by monoclonal proliferation of B lymphocytes resembling those found in the follicular mantle zones. It accounts approximately 2% to 10% of all NHL.^2-8^ Molecular pathogenesis is based on chromosomal translocation t(11;14) (q13; q32) that fuses the immunoglobulin heavy chain enhancer-promoter to the transcription unit of proto-oncogene CCND1, leading to aberrant expression of cyclin D1. The translocation increases expression of cyclin D1, resulting in an alteration in apoptosis and activation of anti-apoptotic pathways like PI3K/Akt (phosphatidylinositol 3-kinases/protein kinase B, also known as Akt) and NF-κB (nuclear factor kappa-light-chain-enhancer of activated B-cells) that causes an abnormal proliferation of MCL cells.^4-6,9^ It has a variable clinical presentation ranging from chronic/subtle to fulminant disease with short survival.^2-4^ The current World Health Organization classification distinguishes aggressive (blastoid and pleomorphic) and other variants (small cell, marginal zone like).^2,3^ MCL is most commonly seen in elderly adults (median age of onset is 60-70 years) and has a predilection to Caucasian males.^2,3^ Most patients present with an advanced stage of the disease, with multiple lymphatic involvement and hepatosplenomegaly, which carries worse prognosis.^2,7^ In some patients, MCL involves extranodal organs, particularly bone marrow, peripheral blood, and gastrointestinal sites,^2,6,9^ which makes it challenging to perform an initial staging and determine the treatment plan. European MCL Network developed a Mantle Cell International Prognostic Index (MIPI) that can be used for risk stratification and allows clinicians to perform a risk-adapted therapy and interventions. MIPI is calculated based on age, Eastern Cooperative Oncology Group performance status, lactate dehydrogenase levels, and WBC count and is considered a useful tool to classify patients with MCL into low-, intermediate-, and high-risk categories.^10^ Therapeutic options are divided into an aggressive approach with high response rates and less aggressive interventions for older patients with poorer performance status based on MIPI score.^2-4^ Multiple front-line therapy regimens are used based on patient characteristics, prognostic classification, and comorbidities. No standard first-line therapy is used in MCL. Most of these regimens control the disease for longer periods, but none of them are curative.

Aggressive treatment includes intensive chemotherapy with R-HyperCVAD/MA, rituximab in combination with alternating cycles of maxi-CHOP (cyclophosphamide, doxorubicin, vincristine, prednisone), and high-dose cytarabine, R-CHOP (rituximab plus cyclophosphamide, doxorubicin, vincristine, and prednisone)/RDHAP (rituximab, cisplatin, cytarabine, dexamethasone) with subsequent autologous hemopoietic stem cell transplant followed by maintenance rituximab every 2 months for 3 years in younger, fit patients.^1^ Older and frail patients will undergo less rigorous strategies with R-CHOP followed by rituximab maintenance, R-bendamustine, or novel agents with promising results.^2-4^ Current observation shows that most of the patients will have good response rates after the first treatment, but relapse is expected in a limited time, making an overall prognosis unfavorable.^2,4^

Non-chemotherapeutic approaches are being investigated while minimizing toxicity to provide prolonged remissions. Multiple clinical trials are evaluating difference combinations—for example, a phase 2 clinical trial evaluating R^2^ regimen (rituximab and lenalidomide) combination for induction followed by maintenance showed 4 year progression-free survival of 69.7% and 4-year overall survival of 82% with one third of the patients remaining in complete remission beyond 5 years.^11^ Currently, a triplet regimen adding ibrutinib to R^2^ regimen is under evaluation in a clinical trial.^1^

CNS involvement in MCL is around 4%; however, the incidence at presentation is less than 1%.^12,13^ Bedotto et al first reported CNS infiltration in MCL in 1986.^14^ High MIPI score, B-symptoms, increased serum lactate dehydrogenase, blastoid variant, high Ki-67, bone marrow involvement, as well as poor performance status are possible risk factors for CNS involvement.^12,13^ Patients with CNS involvement should undergo CSF assessment for cytology and flow cytometry. Clinical manifestation at presentation includes headache, altered mental status, and cranial nerve palsies. Patients at high risk of CNS involvement are advised primary prophylaxis with intrathecal methotrexate and/or cytarabine if the initial chemotherapy regimen did not have CNS penetrating doses of these agents.^12,13^ Bone marrow involvement in MCL ranges from 40% to 80%.^15^ There are biological connections between marrow and CNS involvement in MCL.

Our patient had an atypical initial presentation of MCL with symptomatic involvement of the CNS. The patient was also much younger and did not have any significant lymphadenopathy. His morphological and immunohistochemical studies revealed a rare aberrant CD5 negative subtype of MCL. Based on the review of literature, MCL might occasionally present with predominant extranodal disease, but CNS manifestation as the only symptom is exceedingly rare.

## Conclusion

MCL is a subset of NHL that might occasionally present with sole extranodal manifestation. This disease should be kept in mind even in young patients without significant medical history since it often has an aggressive course with high mortality. Atypical presentation and vague symptoms should prompt all efforts to establish a tissue diagnosis. Our patient also aberrant CD5 negative MCL, which is rare.

As we are learning and understanding more about biology, using risk-adapted treatment strategies along with incorporating novel targeted therapies will be crucial for improving survival and outcomes.

Newer therapies are incorporating Bruton’s tyrosine kinase inhibitors into front-line treatment either as a single agent or in combination with other therapies and using minimal residual disease as prognostic markers to guide treatment decisions.
